# Real‐World Effects of Home‐Based Transcranial Direct Current Stimulation in Depression: A Randomized Controlled Trial of 3‐Week Versus 6‐Week Protocols

**DOI:** 10.1002/brb3.71119

**Published:** 2025-12-10

**Authors:** Hye Yoon Park, Jaesub Park, Daeyoung Roh, Kyungun Jhung, Jhin Goo Chang, Sunyoung Park, Jin Sun Ryu, Gangho Do, Kiwon Lee, Jin Young Park, Woo Jung Kim

**Affiliations:** ^1^ Department of Psychiatry, Yongin Severance Hospital Yonsei University College of Medicine Yongin Republic of Korea; ^2^ Institute of Behavioral Sciences in Medicine Yonsei University College of Medicine Seoul Republic of Korea; ^3^ Department of Psychiatry, Ilsan Paik Hospital Inje University College of Medicine Goyang Republic of Korea; ^4^ Department of Psychiatry, International St. Mary's Hospital Catholic Kwandong University College of Medicine Incheon Republic of Korea; ^5^ Department of Psychiatry Yonsei Forest Mental Health Clinic Seoul Republic of Korea; ^6^ Department of Psychiatry National Health Insurance Corporation Ilsan Hospital Goyang Republic of Korea; ^7^ Department of Psychiatry, Myongji Hospital Hanyang University College of Medicine Goyang Republic of Korea; ^8^ Medical Research Team Digital Medic Co., Ltd. Seoul Republic of Korea; ^9^ Ybrain Research Institute Seongnam‐si Republic of Korea; ^10^ Center For Digital Health, Yongin Severance Hospital Yonsei University Health System Yongin Republic of Korea; ^11^ Institute For Innovation in Digital Healthcare Yonsei University Seoul Republic of Korea; ^12^ Yonsei Graduate Program in Cognitive Science Yonsei University Seoul Republic of Korea

**Keywords:** adverse reactions, cognition, depressive disorder, randomized controlled trials, transcranial direct current stimulation

## Abstract

**Background:**

Despite growing interest in home‐based transcranial direct current stimulation (tDCS) as a scalable treatment for depression, real‐world evidence regarding its effectiveness, cognitive impact, and safety remains limited. Moreover, the optimal stimulation duration for home‐based tDCS has not been clearly established. This study aimed to compare the clinical effects of two home‐based tDCS protocols—one with 3 weeks of active stimulation followed by 3 weeks of sham stimulation (3WA) and another with 6 weeks of active stimulation (6WA)—in patients with major depressive disorder (MDD).

**Methods:**

In this randomized controlled trial, participants diagnosed with MDD were assigned to either the 3WA or 6WA group. Depressive symptoms were measured using the Beck Depression Inventory‐II (BDI) and the Montgomery–Åsberg Depression Rating Scale (MADRS). Cognitive function was assessed with the Digit Symbol Substitution Test (DSST). Adverse events were systematically monitored, and the relationship between psychotropic medication use and adverse event frequency was analyzed using logistic regression.

**Results:**

Both groups showed significant improvements in depressive symptoms and cognitive performance across four assessment points (baseline, Weeks 3, 6, and 12). Linear mixed‐effects models revealed a significant main effect of time for both BDI (*F* = 33.67, *p* < 0.001) and MADRS scores (*F* = 34.50, *p* < 0.001), with no significant group‐by‐time interaction, indicating comparable efficacy between protocols. Model‐derived mean changes from baseline to Week 12 were −7.53 (95% CI −9.85 to −5.21) for BDI and −6.61 (95% CI −8.73 to −4.49) for MADRS. DSST scores also improved significantly over time (*F* = 55.8, *p* < 0.001), with a mean increase of +6.62 points (95% CI +5.17 to +8.05), again showing no significant group difference. Regarding safety, non‐medicated participants reported fewer adverse events, whereas those taking tianeptine experienced significantly more side effects compared with other medication groups.

**Conclusions:**

Both 3‐week and 6‐week active tDCS protocols were associated with improvements in depressive symptoms and cognitive function over time in this naturalistic clinical context; however, as the study did not include a sham control, these changes should be interpreted as comparative rather than causal effects. The 3‐ and 6‐week protocols demonstrated similar therapeutic outcomes, suggesting that shorter courses may be sufficient. These findings support the real‐world applicability of at‐home tDCS and highlight the need to consider concurrent pharmacotherapy when evaluating tolerability and safety.

**Trial Registration:**

ClinicalTrials.gov identifier: NCT05539131

## Graphical abstract

In this randomized controlled trial, both 3‐week and 6‐week home‐based tDCS protocols effectively reduced depressive symptoms, with no significant difference between the two. Cognitive performance, as measured by the DSST, improved over time in both groups. Adverse events were mild and varied by the type of concurrent psychotropic medication.

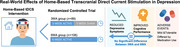

## 1 Introduction

With its rising clinical interest, transcranial direct current stimulation (tDCS) is increasingly recognized as a promising therapeutic option for major depressive disorder (MDD). This noninvasive and generally well‐tolerated modality may complement established pharmacological and psychotherapeutic treatments. The technique involves the application of a low‐intensity electrical current through scalp‐mounted electrodes, producing polarity‐dependent effects—namely, increased cortical excitability under the anode and decreased excitability under the cathode (Nitsche and Paulus 2000). A growing body of research has examined the clinical efficacy of tDCS for depressive episodes, and numerous studies have reported beneficial outcomes (Brunoni et al. 2013; Fregni et al. 2021; Lefaucheur et al. 2017; Razza et al. 2020).

Parallel advancements in device technology have enabled the use of home‐based tDCS for treating MDD (Oh et al. 2023; Woodham et al. 2025), expanding its feasibility in routine psychiatric care. In South Korea, this shift has been particularly notable since the approval of home‐based tDCS using MINDD STIM+ (Ybrain Inc., Gyeonggi‐do, Korea), the first prescription‐based digital neuromodulation device for depression. The device received market authorization from the Ministry of Food and Drug Safety (MFDS) in 2021 and was designated a conditional new health technology by the Ministry of Health and Welfare in June 2022. Its clinical adoption has since grown rapidly, with over 90,000 cumulative prescriptions issued as of September 2024. However, significant knowledge gaps persist regarding the optimal clinical implementation of tDCS, especially the appropriate duration of treatment. Notably, neither international guidelines (Bikson et al. 2023; Fregni et al. 2021; Lefaucheur et al. 2017) nor Korean regulatory materials (Korea National Evidence‐based Healthcare Collaborating Agency 2019) specify the optimal duration of tDCS treatment. Most studies informing these guidelines have employed relatively short intervention periods, typically ranging from 2 to 6 weeks, but no formal consensus on treatment duration has been established.

Beyond its antidepressant effects, tDCS has also been investigated for its potential to improve cognitive functioning. MDD is frequently associated with persistent deficits in domains such as attention, processing speed, and executive function (Culpepper et al. 2017; Wagner et al. 2012). These cognitive impairments often remain unresolved despite standard first‐line treatments, including antidepressant medications, psychotherapy, and electroconvulsive therapy (Bortolato et al. 2016; Shilyansky et al. 2016). The potential cognitive‐enhancing effects of tDCS in MDD have been hypothesized based on its ability to modulate neural activity in the prefrontal cortex, thereby promoting neuroplasticity (Bennabi and Haffen 2018; Chase et al. 2020). However, whereas some studies have reported improvements in cognitive performance following repeated sessions of tDCS (Wang et al. 2024), others have found no significant advantage over sham stimulation (Martin et al. 2018). Considering that cognitive dysfunction significantly contributes to functional disability and poor treatment outcomes in MDD, further investigation is warranted to determine whether tDCS confers reliable cognitive benefits in routine clinical settings.

From a safety perspective, tDCS is generally regarded as a low‐risk and well‐accepted intervention, with only mild and transient side effects, such as skin redness and itching (Bennabi and Haffen 2018). Although its non‐invasive nature and favorable tolerability profile make tDCS an appealing treatment modality, much of the existing research has been conducted under tightly controlled experimental conditions. Such studies often exclude patients with comorbidities or those taking concurrent psychotropic medications, limiting the generalizability of their findings. These uncertainties underscore the need for further research conducted in real‐world clinical settings that better reflect clinical heterogeneity and pharmacological complexity. To address these gaps, the present study evaluated the effectiveness, cognitive impact, and safety of home‐based tDCS in a real‐world outpatient population with MDD. This study compared two stimulation protocols to address the lack of consensus on the optimal duration of tDCS for MDD. To our knowledge, all three randomized controlled trials of home‐based tDCS in MDD conducted prior to this study were limited to 6 weeks of stimulation (Borrione et al. 2024; Kumpf et al. 2023; Oh et al. 2023). Based on this precedent, we adopted the 6‐week active protocol (6WA) as a reference condition. In contrast, the 3‐week active followed by 3‐week sham protocol (3WA) was designed to explore whether a shorter active stimulation period might be sufficient in a real‐world setting, potentially offering comparable benefits with reduced treatment burden. Moreover, we investigated whether concurrent psychotropic medication use influences the frequency of adverse events.

## 2 Methods

### 2.1 Participants

This multicenter study was conducted at five hospitals across South Korea: Catholic Kwandong University International St. Mary's Hospital (CK), Hallym University Chuncheon Sacred Heart Hospital (HL), National Health Insurance Service Ilsan Hospital (IS), Myongji Hospital (MJ), and Yonsei University Yongin Severance Hospital (YS). Participant enrollment began in November 2022 and concluded in July 2024. This trial was prospectively registered at ClinicalTrials.gov (Identifier: NCT05539131; https://clinicaltrials.gov/study/NCT05539131) prior to participant enrollment and was conducted at multiple clinical sites in accordance with the CONSORT guidelines. The completed CONSORT checklist is provided as supporting information.

**FIGURE 1:**
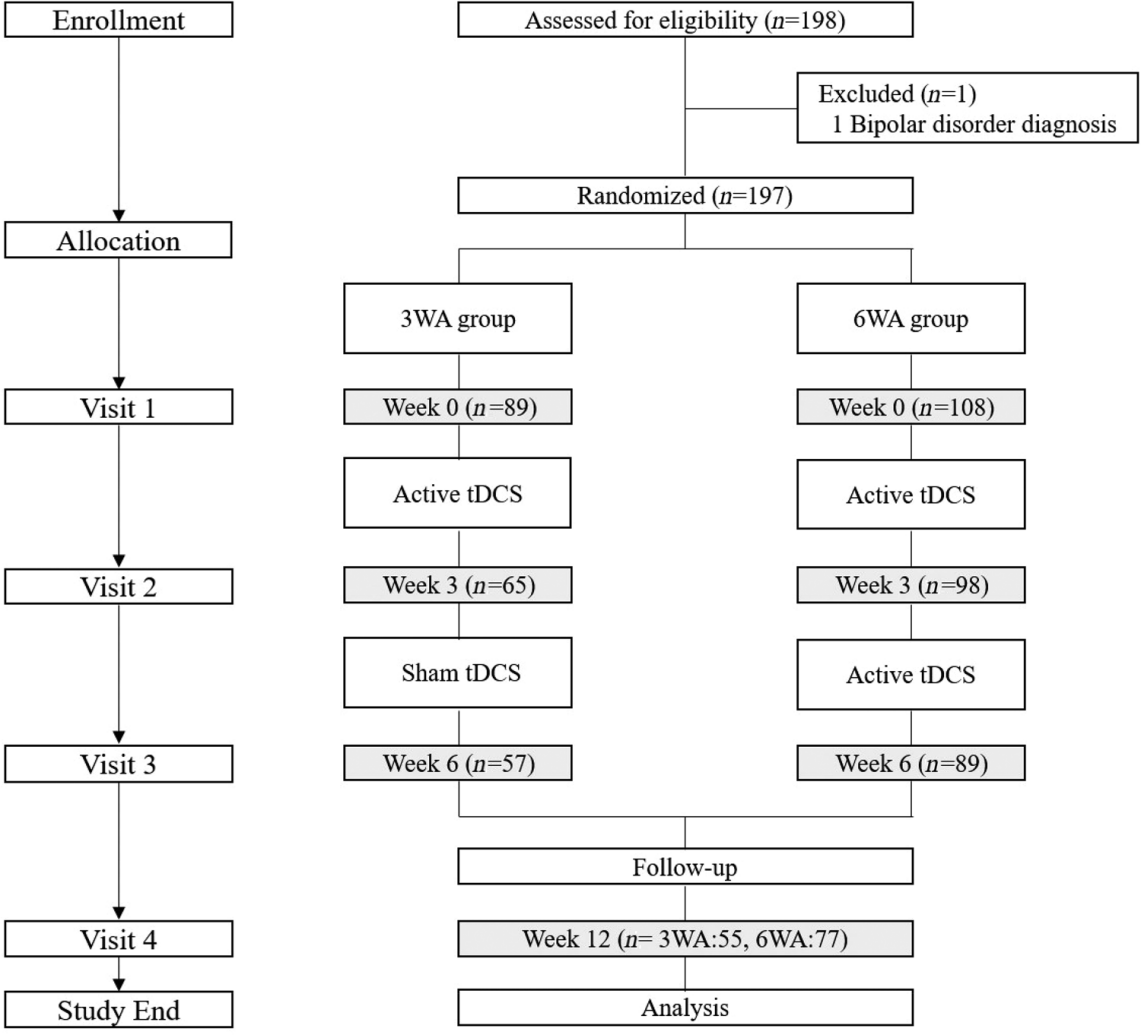
Study flow diagram. Flow of participants through the study phases, including screening, randomization, group allocation, intervention (3WA and 6WA), follow‐up, and analysis. The 3WA group received 3 weeks of active tDCS followed by 3 weeks of sham stimulation, and the 6WA group received 6 consecutive weeks of active tDCS. tDCS, transcranial direct current stimulation.

Participants were recruited through physician referrals and public advertisements. Eligible individuals were adults aged 19–65 years with a primary diagnosis of mild to moderate MDD, as defined by the Diagnostic and Statistical Manual of Mental Disorders, Fifth Edition (DSM‐5) (Association 2013) and confirmed using the Mini International Neuropsychiatric Interview (Sheehan et al. 1998). Only patients with unipolar non‐psychotic depression were included. The severity of depression was operationalized as Beck Depression Inventory‐II (BDI) (Beck et al. 1996) scores between 18 and 28 or Montgomery–Åsberg Depression Rating Scale (MADRS) (Montgomery and Åsberg 1979) scores between 14 and 34 at baseline, corresponding to non‐severe depressive states. The exclusion criteria included a DSM‐5 diagnosis of post‐traumatic stress disorder or obsessive–compulsive disorder; a score > 5 on the suicidal ideation item of the MADRS at any time point; a history of attempted suicide within 6 months prior to screening; scalp conditions (e.g., deformity, inflammation, or other dermatological issues) that interfered with tDCS electrode placement; contraindications to tDCS, such as the presence of metallic implants in the head; and a history of neurological disorders, including epilepsy, stroke, or traumatic brain injury. A summary of the screening, exclusion, and retention process is shown in Figure 1, with detailed dropout reasons listed in Table S1.

### 2.2 Intervention

This study employed a portable tDCS device (MINDD STIM+, Ybrain Inc.) that has been approved by the MFDS of South Korea for at‐home treatment of mild to moderate MDD. The overall structure of the clinical protocol is illustrated in Figure 1. Following the screening process, participants completed four scheduled study visits. At Visit 1 (baseline), participants were randomly assigned to one of two groups: (1) a group receiving 3 weeks of active stimulation followed by 3 weeks of sham stimulation (3WA), or (2) a group receiving 6 consecutive weeks of active stimulation (6WA). Randomization was performed using a computerized sequence, and both participants and outcome assessors were blinded to group allocation (double‐blind design). All tDCS devices were pre‐programmed by an independent researcher not involved in assessments or data analysis. The device interface, sound, and appearance were identical across groups. Participants operated the device at home using uniform instructions, with no visual or operational cues to distinguish active from sham stimulation. In the 3WA group, active stimulation was administered during the first 3 weeks followed by sham stimulation, based on ethical considerations and to reflect real‐world clinical practice in which treatment is typically initiated without delay. Administering sham stimulation first was deemed inappropriate, as it could withhold potentially beneficial treatment from individuals with depression. Between Visit 1 and Visit 2 (Weeks 1–3), both groups received active tDCS sessions at home using the study device. The anode was placed over the F3 region and the cathode over the F4 region, corresponding to the left and right dorsolateral prefrontal cortex, respectively, based on the International 10–20 system for electroencephalography. Each session delivered 1.5–2 mA of current for 30 min, and five sessions were conducted per week. Between Visit 2 and Visit 3 (Weeks 4–6), the 6WA group continued active stimulation, and the 3WA group received sham stimulation. For the sham condition, the device automatically turned off after 1 min of low‐intensity stimulation to mimic the initial physical sensations (e.g., tingling or burning) of active tDCS without providing therapeutic stimulation. This approach is a validated method to maintain participant blinding in tDCS trials. To ensure pharmacological stability during the intervention, only those who agreed not to alter their psychotropic medication regimens from 3 weeks prior to the start of treatment until the end of the 6‐week protocol were included. Use of as‐needed medications was permitted during this period. A follow‐up assessment was conducted at Visit 4, which took place 12 weeks after Visit 3.

### 2.3 Outcome Measures

The co‐primary outcome was the change in depressive symptoms measured by the BDI and MADRS. These two instruments were selected to jointly capture depressive symptom change from both self‐reported and clinician‐rated perspectives, as pre‐specified in the study protocol. Cognitive performance was assessed using the Digit Symbol Substitution Test (DSST) (Wechsler 1981).

### 2.4 Adverse Events Monitoring and Classification

Adverse events were systematically assessed at each study visit through clinician interviews and participant self‐reports. All reported adverse events were categorized into the following clinical domain categories: dermatologic, gastrointestinal, neurologic, psychiatric, cardiovascular, musculoskeletal, and other. Dermatologic events included symptoms such as skin stinging, itching, rash, and urticaria. Gastrointestinal events consisted of appetite loss and nausea, and neurologic events included headache and dizziness. Psychiatric events encompassed agitation, insomnia, worsening of depressive symptoms, and increased suicidal ideation. Cardiovascular (e.g., palpitations), musculoskeletal (e.g., joint pain, muscle cramps), and nonspecific symptoms such as fatigue were also recorded.

### 2.5 Psychotropic Medication Classification

Psychotropic medication use was documented at baseline and monitored at every visit to ensure consistency throughout the intervention phase. Participants were instructed to maintain their prescribed psychiatric medications without changes in type or dosage during the tDCS treatment period. For analytical purposes, medications were classified by into the following categories according to their pharmacologic mechanisms (listed alphabetically): antiepileptic drugs (e.g., divalproex sodium); antipsychotics (e.g., aripiprazole, quetiapine, and risperidone); benzodiazepines (e.g., alprazolam, clonazepam, lorazepam, and diazepam); melatonergic antidepressants (e.g., agomelatine); noradrenergic and specific serotonergic antidepressants (e.g., mirtazapine); norepinephrine–dopamine reuptake inhibitors (e.g., bupropion); serotonergic modulators (e.g., trazodone); serotonin–norepinephrine reuptake inhibitors (SNRIs; e.g., desvenlafaxine, venlafaxine, and duloxetine); selective serotonin reuptake enhancers (SSREs; e.g., tianeptine); selective serotonin reuptake inhibitors (SSRIs; e.g., escitalopram, sertraline, fluoxetine, paroxetine); and tricyclic antidepressants (e.g., doxepin, amitriptyline). Participants were classified as non‐medicated if they were not taking any psychotropic medication at the time of study enrollment. These classifications were used in subgroup analyses to evaluate the associations between the drug class and the occurrence of adverse events. A detailed summary of the psychotropic medications used by participants is provided in Table S3.

### 2.6 Statistical Analysis

All statistical analyses were conducted using IBM SPSS Statistics for Windows, Version 28.0 (IBM Corp., Armonk, NY, USA). The analyses were performed on the intention‐to‐treat (ITT) population, which included all randomized participants with at least one post‐baseline assessment, regardless of protocol adherence or treatment completion. A more complete analysis was also conducted using data from the participants who completed all study visits. The results were consistent with those of the ITT analysis, with no differences in statistical significance. Baseline demographic and clinical characteristics were compared between the two groups using independent‐sample *t*‐tests for continuous variables and chi‐square tests for categorical variables. For the co‐primary outcomes (BDI and MADRS), linear mixed‐effects models (LMMs) were employed to analyze the repeated measures of depressive symptoms over time. Both measures were analyzed independently to evaluate convergence between self‐reported and clinician‐rated changes in depression severity. The models included fixed effects for the group (3WA vs. 6WA), time (Visits 1–4), and group × time interaction, with random intercepts for participants to account for within‐subject correlations. The covariates included age, sex, years of education, and study site. Inclusion of these covariates did not alter the statistical significance of the primary outcomes; therefore, all reported results were gathered from the adjusted models. A secondary outcome analysis was conducted using a similar LMM approach to evaluate the changes in cognitive performance, as measured by the DSST across Visits 1–4. To examine the factors associated with the occurrence of adverse events, we performed binary logistic regression analyses with the presence or absence of adverse events as the dependent variable. Medication status and other relevant covariates were entered as independent variables. This analysis was used to explore the relationship between specific psychotropic drug classes and the likelihood of experiencing adverse events during the intervention period. Two‐sided *p*‐values < 0.05 were considered statistically significant for all analyses.

## 3 Results

### 3.1 Participants

A total of 198 individuals were assessed for eligibility, and one was excluded due to a diagnosis of bipolar disorder. The remaining 197 participants were randomized to either the 3WA group (*n* = 89) or the 6WA group (*n* = 108), as illustrated in Figure 1. Following randomization, participants underwent active ± sham tDCS interventions across 6 weeks, and follow‐up assessments were conducted in Week 12. Dropouts occurred at multiple stages of the trial: By Week 3 (Visit 2), 24 participants in the 3WA group and 10 in the 6WA group had withdrawn. By Week 6 (Visit 3), 57 and 89 participants remained in the 3WA and 6WA groups, respectively. Fifty‐five participants in the 3WA group and 77 in the 6WA group completed the final follow‐up visit in Week 12 (Visit 4). The reasons for dropout—including withdrawal of consent, medical issues, protocol violations, and loss to follow‐up—are detailed in Table S1.

At baseline, the demographic and clinical characteristics were well balanced between the two groups. No significant differences were observed in age, sex distribution, years of education, body mass index, study site, medical or psychiatric comorbidity burden, or overall psychotropic medication load. Full details are provided in Table 1.

**TABLE 1:** General characteristics of the subjects.

Variable	All (*n* = 197)	3WA (*n* = 89)	6WA (*n* = 108)	*t* or *χ* ^2^	*p*‐value
**Age (years)**	36.7 ± 13.3	36.9 ± 14.04	36.6 ± 12.76	0.122	0.903
**Sex (male/female)**	73/124	38/51	35/73	2.97	0.085
**Years of education**	14.5 ± 2.2	14.5 ± 1.95	14.5 ± 2.44	−0.094	0.925
**BMI (kg/m^2^)**	23.9 ± 5.06	24.7 ± 5.83	23.3 ± 4.27	1.856	0.065
**Site (CK/HL/IS/MJ/YS)**	23/30/40/49/55	11/15/20/24/19	12/15/20/25/36	3.519	0.475
**Medical comorbidity burden (*n*)**	0.2 ± 0.43	0.2 ± 0.41	0.3 ± 0.45	−1.045	0.297
**Psychiatric comorbidity burden (*n*)**	0.4 ± 0.64	0.4 ± 0.58	0.5 ± 0.69	−0.86	0.391
**Psychotropic medication load (*n*)**	2.9 ± 1.80	2.9 ± 1.58	2.9 ± 1.98	−0.278	0.781

*Note*: Values are presented as mean ± standard deviation. 3WA, 3‐week active followed by 3‐week sham stimulation group; 6WA, 6‐week active stimulation group; BMI, body mass index; CK, Catholic Kwandong University International St. Mary's Hospital; HL, Hallym University Chuncheon Sacred Heart Hospital; IS, National Health Insurance Service Ilsan Hospital; MJ, Myongji Hospital; YS, Yongin Severance Hospital. Medical/psychiatric comorbidity burden refers to the number of concurrent medical or psychiatric diagnoses. Psychotropic medication load refers to the number of currently prescribed psychotropic agents.

### 3.2 Depression Symptom Outcomes

**FIGURE 2:**
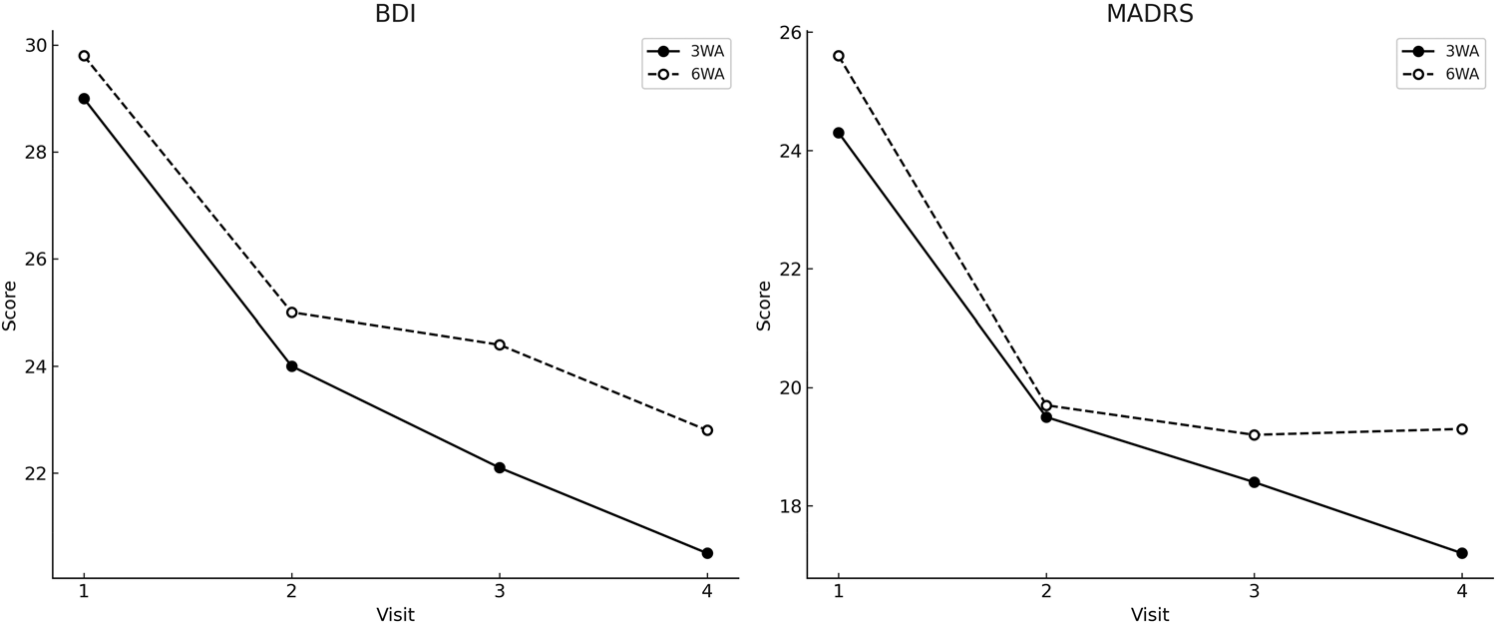
Changes in depressive symptom scores across visits by group. Line plots showing changes in BDI and MADRS scores across four study visits in the 3WA and 6WA groups. Both groups demonstrated significant improvement in depressive symptoms over time. No significant group difference or group × time interaction was found in the linear mixed‐effects model analysis. 3WA, 3‐week active followed by 3‐week sham stimulation; 6WA, 6‐week active stimulation; BDI, Beck Depression Inventory‐II; MADRS, Montgomery–Åsberg Depression Rating Scale.

The primary outcome was the change in depressive symptoms, assessed by BDI and MADRS scores across Visits 1–4. Both groups showed significant improvements in depressive symptoms over time. LMM analysis revealed a significant main effect of time for both BDI (*F* = 33.671, *p* < 0.001) and MADRS scores (*F* = 34.499, *p* < 0.001), indicating symptom improvement across the study period. Model‐derived mean changes from baseline to Week 12 were −7.53 (95% CI −9.85 to −5.21) for BDI and −6.61 (95% CI −8.73 to −4.49) for MADRS. However, there were no significant main effects of the group (BDI: *p* = 0.365; MADRS: *p* = 0.511) or group × time interaction (BDI: *p* = 0.862; MADRS: *p* = 0.662), suggesting no differential effect between the 3WA and 6WA protocols (Table 2; Figure 2). A more complete analysis, limited to participants who completed all four visits, yielded results consistent with those of the ITT analysis. The pattern of significant time effects without group‐by‐time interaction remained unchanged. Study site was included as a covariate in the statistical models, and no significant main or interaction effects involving site were observed for any of the primary or secondary outcomes.

**TABLE 2:** Changes in BDI and MADRS scores by group across time points and results from the linear mixed‐effects model analysis.

		Scores by visit				LMM analysis results			
Measure	Group	Visit 1	Visit 2	Visit 3	Visit 4	Time	Group	Time × group
**BDI**	3WA	28.91 ± 10.98	23.98 ± 13.32	22.09 ± 13.66	20.49 ± 14.46	33.671 (<0.001)	0.825 (0.365)	0.249 (0.862)
	6WA	29.81 ± 12.88	24.96 ± 13.01	24.35 ± 13.85	22.81 ± 14.91			
**MADRS**	3WA	24.29 ± 7.71	19.43 ± 8.79	18.42 ± 10.91	17.13 ± 12.95	34.499 (<0.001)	0.434 (0.511)	0.529 (0.662)
	6WA	25.63 ± 8.15	19.76 ± 8.40	19.15 ± 9.26	19.30 ± 10.35			

*Note*: Scores by visit are presented as mean ± standard deviation. The LMM analysis results refer to the *F*‐statistics and associated *p*‐values derived from linear mixed‐effects models (LMMs), including fixed effects for time, group, and their interaction.

Abbreviations: 3WA, 3‐week active followed by 3‐week sham stimulation; 6WA, 6‐week active stimulation; BDI, Beck Depression Inventory‐II; MADRS, Montgomery–Åsberg Depression Rating Scale.

### 3.3 Cognitive Function

**FIGURE 3:**
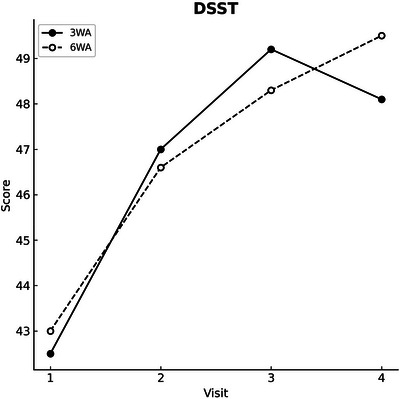
Changes in cognitive performance across visits by group. Line plot depicting changes in DSST scores across four study visits in the 3WA and 6WA groups. Both groups showed significant improvement in cognitive performance over time. No significant group difference or group × time interaction was found in the linear mixed‐effects model analysis. DSST, Digit Symbol Substitution Test; 3WA, 3‐week active followed by 3‐week sham stimulation; 6WA, 6‐week active stimulation.

Cognitive performance, as measured by the DSST, significantly improved over time (*F* = 55.754, *p* < 0.001), with both groups demonstrating similar trajectories. Model‐derived mean improvement from baseline to Week 12 was +6.62 points (95% CI +5.17 to +8.05). No significant group effect (*p* = 0.617) or group × time interaction (*p* = 0.354) was observed, indicating comparable cognitive gains in both groups (Table 3; Figure 3). The completer analysis of participants who completed all four visits showed results consistent with the ITT analysis. The time effects remained significant, and no group‐related differences emerged, confirming the robustness of the observed cognitive improvements.

**TABLE 3:** Changes in DSST scores by group across time points and results from the linear mixed‐effects model analysis.

		Scores by visit				LMM analysis results			
Measure	Group	Visit 1	Visit 2	Visit 3	Visit 4	Time	Group	Time × group
**DSST**	3WA	42.47 ± 11.60	46.97 ± 10.08	49.11 ± 10.46	48.09 ± 10.54	55.754 (<0.001)	0.250 (0.617)	1.089 (0.354)
	6WA	42.99 ± 10.93	46.64 ± 10.95	48.31 ± 11.64	49.51 ± 12.10			

*Note*: Scores by visit are presented as mean ± standard deviation. The LMM analysis results refer to the *F*‐statistics and associated *p*‐values derived from linear mixed‐effects models (LMMs), including fixed effects for time, group, and their interaction.

Abbreviations: 3WA, 3‐week active followed by 3‐week sham stimulation; 6WA, 6‐week active stimulation; DSST, Digit Symbol Substitution Test.

### 3.4 Adverse Events and Psychotropic Medication Use

A total of 76 adverse events were reported during the study. The most frequent events were dermatologic in nature, particularly skin stinging (*n* = 40) and itching (*n* = 11), followed by neurologic (*n* = 8), psychiatric (*n* = 7), and other categories (Table S2). Most adverse events were mild and transient. At baseline, most participants were taking psychotropic medications; only 15 individuals were classified as non‐medicated. Full details of the medication types and distribution are provided in Table S3.

**FIGURE 4:**
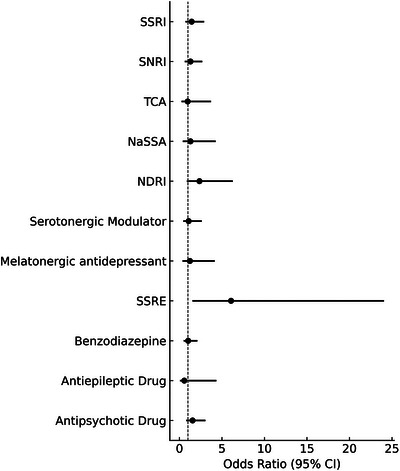
Odds ratios of adverse events by psychotropic medication class. Odds ratios and 95% confidence intervals for the likelihood of adverse events associated with each class of psychotropic medication. Results are derived from a binary logistic regression model. NaSSA, noradrenergic and specific serotonergic antidepressant; NDRI, norepinephrine–dopamine reuptake inhibitor; SNRI, serotonin–norepinephrine reuptake inhibitor; SSRE, selective serotonin reuptake enhancer; SSRI, selective serotonin reuptake inhibitor; TCA, tricyclic antidepressant.

Logistic regression revealed that non‐medicated participants had a significantly lower risk of adverse events (OR = 0.187, 95% CI: 0.039–0.900, *p* = 0.037), as shown in Table 4. Among those taking psychotropic medications, only tianeptine (an SSRE) was significantly associated with increased adverse events (OR = 6.073, 95% CI: 1.533–24.059, *p* = 0.01), as detailed in Table 5 and illustrated in Figure 4. The adverse event occurrence was not significantly associated with any other class of antidepressants or with benzodiazepines, antipsychotics, or antiepileptic drugs.

**TABLE 4:** Logistic regression analysis of adverse effects in non‐medicated individuals.

Variable	*B* (SE)	Wald	*p*‐value	Odds ratio (95% CI)
**Non‐medicated**	−1.679 (0.803)	4.371	0.037	0.187 (0.039–0.900)
**Age**	0.009 (0.011)	0.613	0.434	1.009 (0.987–1.031)
**Sex**	0.368 (0.312)	1.396	0.237	1.445 (0.785–2.661)
**Education years**	0.077 (0.068)	1.268	0.26	1.080 (0.945–1.234)
**Site**	−0.007 (0.115)	0.004	0.948	0.993 (0.792–1.243)
**Constant**	−2.255 (1.200)	3.528	0.06	0.105 (−)

*Note*: Results from a binary logistic regression model predicting the occurrence of adverse events in non‐medicated participants. “Site” refers to the study location variable.

Abbreviations: *B*, unstandardized regression coefficient; CI, confidence interval; SE, standard error; Wald, Wald test statistic.

**TABLE 5:** Logistic regression analysis of adverse effects associated with current psychotropic medication use.

Variable	*B* (SE)	Wald	*p*‐value	Odds ratio (95% CI)
**SSRI**	0.364 (0.359)	1.03	0.31	1.440 (0.712–2.910)
**SNRI**	0.264 (0.369)	0.514	0.473	1.303 (0.632–2.683)
**TCA**	−0.029 (0.685)	0.002	0.966	0.972 (0.254–3.721)
**NaSSA**	0.267 (0.604)	0.196	0.658	1.306 (0.400–4.267)
**NDRI**	0.855 (0.501)	2.908	0.088	2.352 (0.880–6.284)
**Serotonergic modulator**	0.085 (0.451)	0.036	0.85	1.089 (0.450–2.637)
**Melatonergic**	0.212 (0.619)	0.117	0.732	1.236 (0.367–4.160)
**SSRE**	1.804 (0.702)	6.594	0.01	6.073 (1.533–24.059)
**Benzodiazepine**	0.035 (0.365)	0.009	0.924	1.035 (0.507–2.115)
**Antiepileptic drug**	−0.560 (1.036)	0.293	0.589	0.571 (0.075–4.346)
**Antipsychotic drug**	0.441 (0.345)	1.632	0.201	1.554 (0.790–3.058)
**Age**	0.010 (0.013)	0.605	0.437	1.010 (0.985–1.036)
**Sex**	0.450 (0.331)	1.845	0.174	1.568 (0.819–3.001)
**Education years**	0.069 (0.076)	0.831	0.362	1.071 (0.924–1.243)
**Group**	−0.325 (0.319)	1.035	0.309	0.723 (0.387–1.351)
**Site**	0.077 (0.124)	0.385	0.535	1.080 (0.846–1.379)
**Constant**	−3.287 (1.354)	5.893	0.015	0.037 (−)

*Note*: Results from a binary logistic regression model evaluating the association between current psychotropic medication use and the occurrence of adverse events. Medication classes include SSRI (selective serotonin reuptake inhibitor), SNRI (serotonin–norepinephrine reuptake inhibitor), TCA (tricyclic antidepressant), NaSSA (noradrenergic and specific serotonergic antidepressant), NDRI (norepinephrine–dopamine reuptake inhibitor), SSRE (selective serotonin reuptake enhancer), and other psychotropic categories.

Abbreviations: *B*, unstandardized regression coefficient; CI, confidence interval; SE, standard error; Wald, Wald test statistic.

## 4 Discussion

To our knowledge, this is the first study to comprehensively evaluate the real‐world effectiveness, cognitive impact, and safety of at‐home tDCS in patients with mild to moderate MDD while also examining the influence of concurrent psychotropic medication use. Both tDCS protocols (3WA and 6WA) led to significant improvements in depressive symptoms over time, with no significant differences between the groups. Cognitive enhancement was consistently observed across sessions, as reflected in the increased DSST scores. Adverse events were generally mild and most commonly dermatologic. Subgroup analysis revealed that non‐medicated participants reported significantly fewer adverse events compared with medicated participants, whereas only tianeptine use was significantly associated with a higher risk of adverse events. These findings collectively support the comparable clinical outcomes of the 3‐ and 6‐week home‐based tDCS protocols and highlight the need to account for concurrent psychotropic medications when evaluating their safety and tolerability.

The significant reduction in depressive symptoms observed across both groups aligns with the substantial body of literature supporting the antidepressant efficacy of tDCS. Meta‐analyses and randomized controlled trials have demonstrated that tDCS applied over the dorsolateral prefrontal cortex can lead to meaningful reductions in depressive symptoms, particularly when administered in structured, repeated sessions (Brunoni et al. 2017; Moffa et al. 2020; Razza et al. 2020). In our real‐world outpatient study, symptom improvement was consistently observed across both self‐rated (BDI) and clinician‐rated (MADRS) measures, reinforcing the reliability and clinical validity of the findings. Importantly, no significant differences were found between the 3‐week and 6‐week active stimulation protocols (3WA vs. 6WA), suggesting that a shorter course of tDCS may be sufficient to achieve therapeutic benefit. To our knowledge, no prior study has directly compared different durations of active tDCS. One recent study by Nikolin et al. (2023) analyzing individual participant data from 10 randomized controlled trials suggested that the antidepressant effects of active tDCS tend to peak approximately 6 weeks after treatment initiation, which corresponds to the time when symptom severity typically reaches its lowest point. Although this finding provides insight into the temporal dynamics of tDCS responses, it was not derived from a randomized comparison of stimulation durations and should be interpreted accordingly. The 2021 MFDS approval of the first portable, at‐home tDCS device for mild and moderate depression (Bikson et al. 2023; Park et al. 2020) opened a new avenue for decentralized neuromodulation. Our findings add empirical support for its clinical utility and suggest that a pragmatic, remotely supervised protocol may be both effective and scalable in routine care. By simulating the conditions of routine outpatient care, this study contributes to a more practice‐oriented understanding of tDCS and strengthens the evidence base for its broader implementation as a digital therapeutic for depressive disorders.

In addition to symptom improvement, participants in both groups demonstrated significant gains in cognitive performance over time, as measured by the DSST. Numerous studies have examined the cognitive domains affected by tDCS in patients with MDD, including executive function (Ciullo et al. 2021; Huang et al. 2023), working memory (Murphy et al. 2023; Salehinejad et al. 2015), processing speed (Gögler et al. 2017), and verbal learning and memory (McClintock et al. 2020). The DSST, used in the present study, is particularly sensitive to changes in processing speed, attention, psychomotor efficiency, and, to some extent, executive function (Benedict et al. 2017; Berrigan et al. 2022; Salthouse 1992; Smith 1973), which are frequently impaired in individuals with depression (Douglas et al. 2018; Varghese et al. 2022). Therefore, the observed improvement in DSST scores supports the notion that tDCS may facilitate enhancement in these cognitive domains. However, the efficacy of tDCS for cognitive enhancement in MDD may be influenced by various factors, including stimulation parameters and genetic variability (McClintock et al. 2020). Future clinical effectiveness trials incorporating these moderators are warranted to better elucidate the mechanisms by which tDCS exerts its cognitive benefits. From a clinical perspective, these findings highlight the potential utility of tDCS not only for alleviating mood symptoms but also for targeting cognitive deficits in depression, which substantially contribute to functional disability and treatment resistance.

The adverse events reported during the tDCS intervention were generally mild and transient. Skin‐related symptoms, such as stinging and itching, were the most commonly reported. This finding is consistent with previous literature indicating that the common adverse effects of tDCS include itching, tingling, burning sensations, and skin redness (Brunoni et al. 2011; Chhabra et al. 2020; Turnbull et al. 2023). Notably, the subgroup analysis revealed that non‐medicated participants were significantly less likely to report adverse events, suggesting that concurrent psychotropic medication use may increase susceptibility to tDCS‐related discomfort. Among the various medication classes analyzed, only the use of tianeptine was significantly associated with an increased risk of adverse events. Several pharmacological mechanisms may underlie this finding. First, tianeptine exhibits an atypical pharmacodynamic profile; unlike most antidepressants, it does not inhibit monoamine reuptake but rather enhances serotonin reuptake, in contrast to the mechanism of SSRIs and SNRIs (Alamo et al. 2019; Kato and Weitsch 1988; McEwen et al. 2010). This action may sensitize serotonergic pathways to the neuromodulatory effects of tDCS, increasing the likelihood of somatosensory side effects. Second, recent studies have highlighted tianeptine's broader, multimodal pharmacology, including its modulation of glutamatergic transmission through inhibition of NMDA receptors and indirect effects on AMPA receptors (Bailey et al. 2017; McEwen et al. 2010). Given that tDCS also modulates cortical excitability partly through glutamatergic pathways, co‐administration with tianeptine may result in synergistic or overstimulating effects on local excitatory neurotransmission or neurovascular responses. Third, it is also plausible that patients prescribed tianeptine represent a clinically distinct subgroup, potentially with greater somatic sensitivity or prior treatment resistance, which may increase their tendency to perceive or report adverse events. To the best of our knowledge, this is the first study to explore the association between concurrent psychotropic medication use and the occurrence of tDCS‐related adverse events. These findings underscore the importance of considering individual pharmacologic profiles when implementing tDCS in clinical practice, particularly in home‐based settings. Further research is warranted to elucidate drug–stimulation interactions and identify patient‐level predictors of tDCS tolerability.

Several limitations of this study should be acknowledged. First, because the primary aim was to evaluate the effectiveness of tDCS in a real‐world clinical setting, we intentionally did not include an artificial control group, such as a non‐treatment arm or a delayed active stimulation condition. Although this pragmatic design enhances ecological validity, it limits our ability to distinguish the effects of tDCS from natural symptom fluctuations or placebo responses. Second, the assessment of cognitive function was restricted to a single measure (DSST), which may not fully capture the range of cognitive domains affected by depression or modulated by tDCS. Future studies should consider incorporating a more comprehensive battery of neurocognitive assessments to evaluate more diverse domains. Third, differences in the number of completed tDCS sessions and participant attrition over time may have influenced the degree of treatment exposure. However, our LMM analyses yielded consistent results even when restricted to participants with complete data across all visits, suggesting that visit‐to‐visit variation was at least partially controlled. Nonetheless, this limitation should be considered when interpreting the generalizability and robustness of the findings.

In summary, this study demonstrates that at‐home tDCS is a feasible intervention showing comparable improvements across 3‐ and 6‐week protocols; however, efficacy relative to no‐treatment or sham conditions cannot be determined. Importantly, no significant differences were observed between the 3‐week and 6‐week stimulation protocols in reducing depressive symptoms and improving cognitive performance in patients with mild to moderate MDD. The findings support the clinical potential of short‐term remotely supervised neuromodulation as a scalable treatment option, particularly in real‐world outpatient settings. In further support of this, despite moderate attrition rates of 38.2% in the 3WA group and 28.7% in the 6WA group, the majority of participants successfully completed the 12‐week protocol. Given that participants self‐administered stimulation and managed adherence remotely, these findings underscore the feasibility and acceptability of home‐based tDCS in naturalistic clinical settings. Moreover, the observed association between psychotropic medication use—particularly tianeptine—and increased adverse event reporting highlights the need for personalized approaches to tDCS application. Future research should aim to clarify the pharmacodynamic mechanisms underlying such interactions, expand cognitive assessment tools to capture a wider breadth of domains, and examine the long‐term durability of treatment effects.

## Author Contributions

 .

## Ethics Statement

The study protocol was reviewed and approved by the Korean Ministry of Food and Drug Safety (MFDS), as well as the institutional review boards of all participating institutions. All procedures involving human participants were conducted in accordance with the ethical standards of the institutional and national research committees and with the 1964 Declaration of Helsinki and its later amendments.

## Consent

All participants provided written informed consent prior to participation.

## Funding Information

This work was supported by a grant from the Korea Health Technology R&D Project through the Korea Health Industry Development Institute (KHIDI), funded by the Ministry of Health and Welfare, Republic of Korea (grant number: HI22C0520 and RS‐2024‐00439193), and by a grant from the National Research Foundation of Korea (NRF), funded by the Korean government (MSIT) (grant number: RS‐2024‐00458451).

## Conflicts of Interest

The authors declare no conflicts of interest.

## Supporting information




**Supplementary Tables**: brb371119‐sup‐0001‐Tables.pdf

**Supplementary Materials**: brb371119‐sup‐0002‐SuppMat.docx

## Data Availability

The datasets generated and analyzed during the current study are available from the corresponding author on reasonable request.
