# Supplementary material for: Real‐World Effects of Home‐Based Transcranial Direct Current Stimulation in Depression: A Randomized Controlled Trial of 3‐Week Versus 6‐Week Protocols
Source: Brain Behav. 2025 Dec 10;15(12):e71119. doi: 10.1002/brb3.71119 (PMC12696033; doi:10.1002/brb3.71119)
Supplement: Supplementary file 1 — Supplementary Tables: brb371119‐sup‐0001‐Tables.pdf [file BRB3-15-e71119-s001.pdf]

# Supplementary Material

**Supplementary Table 1. Number of Dropouts and Reasons per Visit**

| Visit | 3WA | 6WA |
|---|---|---|
| **Visit 2** | Withdrawal of consent:17<br>Medical withdrawal for safety reasons:5<br>Investigator withdrawal due to study concerns:1<br>Lost to follow-up:1 | Withdrawal of consent:4<br>Medical withdrawal for safety reasons:3<br>Lost to follow-up:1<br>protocol violation:2 |
| **Visit 3** | Withdrawal of consent:2<br>Medical withdrawal for safety reasons:1<br>Lost to follow-up:1<br>protocol violation:4 | Withdrawal of consent:4<br>Medical withdrawal for safety reasons:2<br>protocol violation:3 |
| **Visit 4** | Withdrawal of consent:1<br>protocol violation:1 | Withdrawal of consent:10<br>protocol violation:2 |

Values indicate the number of participants who discontinued the study at each visit, categorized by reason for dropout. 3WA, 3-week active followed by 3-week sham stimulation; 6WA, 6-week active stimulation. Reasons for dropout include withdrawal of consent, medical withdrawal for safety reasons, investigator-initiated withdrawal, loss to follow-up, and protocol violations.

**Supplementary Table 2. Summary of Adverse Event Frequencies by Category**

| Category | n | Reported symptoms |
|---|---|---|
| Skin | 56 | Skin stinging: 40<br>Skin itching: 11<br>Rash: 4<br>Urticaria: 1 |
| Gastrointestinal | 2 | Loss of appetite: 1<br>Nausea: 1 |
| Neurologic | 8 | Headache: 6<br>Dizziness: 2 |
| Psychiatric | 7 | Agitation: 3<br>Insomnia: 2<br>Depressive symptoms: 1<br>Increased suicidal tendency: 1 |
| Cardiovascular | 1 | Palpitations: 1 |
| Musculoskeletal | 3 | Joint Pain: 2<br>Muscle cramps: 1 |
| Others | 5 | Fatigue: 5 |

Values represent the number of participants who reported adverse events within each symptom category. “n” refers to the number of individuals who experienced at least one event in the given category.

**Supplementary Table 3. Summary of Psychotropic Drug Usage Among Participants**

| Class | n | Medications |
|---|---|---|
| Non-medicated | 15 | |
| SSRI | 113 | Escitalopram: 56<br>Sertraline: 23<br>Fluoxetine: 21<br>Vortioxetine: 12<br>Paroxetine: 10 |
| SNRI | 60 | Desvenlafaxine: 31<br>Venlafaxine: 20<br>Duloxetine: 7<br>Milnacipran: 3 |
| TCA | 11 | Doxepine: 9<br>Amitriptyline: 2 |
| NaSSA | 19 | Mirtazapine: 19 |
| NDRI | 24 | Bupropion: 24 |
| Serotonergic modulator | 31 | Trazodone: 31 |
| Melatonergic | 14 | Agomelatine: 14 |
| SSRE | 16 | Tianeptine: 16 |
| Benzodiazepine | 131 | Alprazolam: 79<br>Clonazepam: 57<br>Lorazepam: 26<br>Etizolam: 12<br>Diazepam: 7<br>Bromazepam: 2<br>Flunitrazepam: 2<br>Triazolam: 2<br>Clobazam: 1 |
| Antiepileptic drug | 5 | Divalproex sodium: 5 |
| Antipsychotic drug | 68 | Aripiprazole: 41<br>Quetiapine: 33<br>Olanzapine: 1<br>Risperidone: 1<br>Chlorpromazine: 1<br>Perphenazine: 1<br>Amisulpride: 1 |

Values represent the number of participants prescribed medications within each pharmacological class. “n” refers to the number of individuals taking at least one agent from that class. Totals may exceed the number of participants because some individuals were prescribed medications from multiple categories. SSRI, selective serotonin reuptake inhibitor; SNRI, serotonin-norepinephrine reuptake inhibitor; TCA, tricyclic antidepressant; NaSSA, noradrenergic and specific serotonergic antidepressant; NDRI, norepinephrine-dopamine reuptake inhibitor; SSRE, selective serotonin reuptake enhancer.
