# Supplementary material for: Real‐World Effects of Home‐Based Transcranial Direct Current Stimulation in Depression: A Randomized Controlled Trial of 3‐Week Versus 6‐Week Protocols
Source: Brain Behav. 2025 Dec 10;15(12):e71119. doi: 10.1002/brb3.71119 (PMC12696033; doi:10.1002/brb3.71119)
Supplement: Supplementary file 2 — Supplementary Materials: brb371119‐sup‐0002‐SuppMat.docx [file BRB3-15-e71119-s002.docx]

**CONSORT 2010 Checklist of Information to Include When Reporting a Randomized Trial**

| Section/Topic | Item No. | Checklist item | Reported on Page/Section |
| --- | --- | --- | --- |
| Title and abstract | 1a | Identification as a randomized trial in the title | Title page |
|  | 1b | Structured summary of trial design, methods, results, and conclusions | Abstract |
| Introduction | 2a | Scientific background and explanation of rationale | Introduction |
|  | 2b | Specific objectives or hypotheses | End of Introduction |
| Methods | 3a | Description of trial design (e.g., parallel, factorial), including allocation ratio | Section 2.2 Intervention |
|  | 3b | Important changes to methods after trial commencement, with reasons | Not applicable |
| Participants | 4a | Eligibility criteria for participants | Section 2.1 Participants |
|  | 4b | Settings and locations where the data were collected | Section 2.1 Participants |
| Interventions | 5 | The interventions for each group with sufficient details to allow replication | Section 2.2 Intervention |
| Outcomes | 6a | Completely defined pre-specified primary and secondary outcome measures | Section 2.3 Outcome Measures |
|  | 6b | Any changes to trial outcomes after the trial commenced | Not applicable |
| Sample size | 7a | How sample size was determined | Not explicitly stated |
|  | 7b | Explanation of any interim analyses and stopping guidelines | Not applicable |
| Randomization — Sequence generation | 8a | Method used to generate the random allocation sequence | Section 2.2 Intervention |
|  | 8b | Type of randomization; details of any restriction | Section 2.2 Intervention |
| Allocation concealment mechanism | 9 | Mechanism used to implement random allocation sequence | Section 2.2 Intervention |
| Implementation | 10 | Who generated the allocation sequence, enrolled participants, and assigned participants | Section 2.2 Intervention |
| Blinding (masking) | 11a | Who was blinded after assignment to interventions and how | Section 2.2 Intervention |
|  | 11b | Description of the similarity of interventions | Section 2.2 Intervention |
| Statistical methods | 12a | Statistical methods used to compare groups for primary and secondary outcomes | Section 2.6 Statistical Analysis |
|  | 12b | Methods for additional analyses, such as subgroup analyses and adjusted analyses | Section 2.6 Statistical Analysis |
| Results | 13a | Participant flow (numbers randomly assigned, received intended treatment, and analyzed) | Section 3.1 Participants; Figure 1 |
|  | 13b | For each group, losses and exclusions after randomization, with reasons | Section 3.1; Supplementary Table 1 |
| Recruitment | 14a | Dates defining the periods of recruitment and follow-up | Section 2.1 Participants |
|  | 14b | Why the trial ended or was stopped | Not applicable |
| Baseline data | 15 | Baseline demographic and clinical characteristics | Table 1 |
| Numbers analyzed | 16 | Number of participants analyzed in each group | Section 3.1 and Figure 1 |
| Outcomes and estimation | 17a | Results for each primary and secondary outcome, with estimated effect size and precision | Section 3.2–3.3; Tables 2–3; Figures 2–3 |
|  | 17b | For binary outcomes, presentation of both absolute and relative effect sizes | Section 3.4; Table 5 |
| Ancillary analyses | 18 | Results of other analyses (e.g., subgroup analyses) | Section 3.4 |
| Harms | 19 | All important harms or unintended effects | Section 3.4; Supplementary Table 2 |
| Discussion | 20 | Trial limitations | Section 4, paragraph 5 |
|  | 21 | Generalizability (external validity) | Section 4 |
|  | 22 | Interpretation consistent with results and balanced | Section 4 Conclusion |
| Other information | 23 | Registration number and name of trial registry | Title page and Methods (NCT05539131) |
|  | 24 | Where the full trial protocol can be accessed | Title page and Methods (https://clinicaltrials.gov/  study/NCT05539131) |
|  | 25 | Sources of funding and role of funders | Title page |
